# Elevated Plasma Endothelin-1 Levels in Normal Tension Glaucoma and Primary Open-Angle Glaucoma: A Meta-Analysis

**DOI:** 10.1155/2016/2678017

**Published:** 2016-11-14

**Authors:** Shengjie Li, Aiping Zhang, Wenjun Cao, Xinghuai Sun

**Affiliations:** ^1^Department of Clinical Laboratory, Eye & ENT Hospital, Shanghai Medical College, Fudan University, Shanghai, China; ^2^Department of Ophthalmology & Visual Science, Eye & ENT Hospital, Shanghai Medical College, Fudan University, Shanghai, China; ^3^State Key Laboratory of Medical Neurobiology, Institutes of Brain Science, Fudan University, Shanghai, China; ^4^Key Laboratory of Myopia, Ministry of Health, Fudan University, Shanghai, China; ^5^Shanghai Key Laboratory of Visual Impairment and Restoration, Fudan University, Shanghai, China

## Abstract

*Purpose*. The aim of this meta-analysis was to clarify the association between the plasma endothelin-1 level and the risks of normal tension glaucoma (NTG) and primary open-angle glaucoma (POAG).* Methods*. Relevant publications were collected from three databases including PubMed, EMBASE, and the Web of Science through December 31, 2015. In this study, the terms “(endothelin OR ET) AND glaucoma” were searched. Review Manager 5.2 was used to process the data.* Results*. Seven studies (212 cases, 164 controls) were included for the NTG analysis. The mean plasma endothelin-1 level in the NTG subjects was 0.60 pg/mL (*p* = 0.02, 95% CI: 0.17–1.04) higher than that of the healthy controls. Six studies (160 cases, 174 controls) were included for the POAG analysis, and the endothelin-1 level was 0.63 pg/mL (*p* = 0.007, 95% CI: 0.12–1.15) higher in the POAG subjects than in the healthy controls. Additionally, two studies influenced the meta-analysis results regarding the association of plasma endothelin-1 with POAG by sensitivity analysis, and the probability of publication bias was low.* Conclusions*. The observation that NTG and POAG subjects showed significantly elevated endothelin-1 plasma concentrations suggests that a higher plasma level of endothelin-1 might increase the risk of NTG and POAG development.

## 1. Introduction

Glaucoma is a group of heterogeneous diseases characterized by the following features: optic nerve head cupping, glaucomatous visual field defects, and elevated intraocular pressure (IOP) [1]. Open-angle glaucoma (OAG), which is the most common type of glaucoma, is divided into primary open-angle glaucoma (POAG) (IOP > 21 mm Hg) and normal tension glaucoma (NTG) (untreated IOP ≤ 21 mm Hg) based on IOP. The proportion of NTG among OAG varies depending on different population studies: Asian (52%–92%), South African (57.1%), American (31.7%), Netherlandish (38.9%), and Italian (30%) [2]. In OAG patients, irreversible loss of peripheral vision frequently is associated with a pathologic elevation of IOP. Although increased IOP is the most important known risk factor for POAG [3], some other unknown risk factors also exist, especially in patients with NTG [4]. NTG patients who have a lower tolerance compared with POAG may have a higher prevalence of vascular abnormalities. The prevalence of vascular dysregulation is higher in those with NTG than in those with POAG [5, 6]. Over the last few years, several studies suggested that vascular dysfunction might play an important role in the pathogenesis of GON (glaucomatous optic neuropathy) [7, 8]. Endothelin-1 (ET-1), being one of the key regulators for vascular function and widely expressed by endothelia cells [9], has been hypothesized to play important role in the progression of GON [10, 11].

Although many publications evaluated the plasma ET-1 level in NTG and POAG, the association between plasma ET-1 and glaucoma still remained controversial. Several studies showed that plasma ET-1 level is significantly elevated in NTG [12–16] and POAG [12, 15] groups than in control group while others reported that no significant difference of ET-1 levels was observed between those three groups [13, 17–19]. Therefore, the aim of this meta-analysis was to evaluate whether the plasma ET-1 level is significantly elevated in NTG and POAG and to confirm the association between the plasma ET-1 level and glaucoma.

## 2. Materials and Methods

### 2.1. Literature Search Strategy

The studies were obtained from PubMed, EMBASE, and the Web of Science. The search terms “(endothelin-1 OR ET-1) AND glaucoma” were used to retrieve the relevant studies. The reference lists of all the relevant articles were searched to identify other studies. We searched for relevant studies from the beginning of indexing for each database to August 31, 2015.

### 2.2. Inclusion Criteria

**Figure 1:**
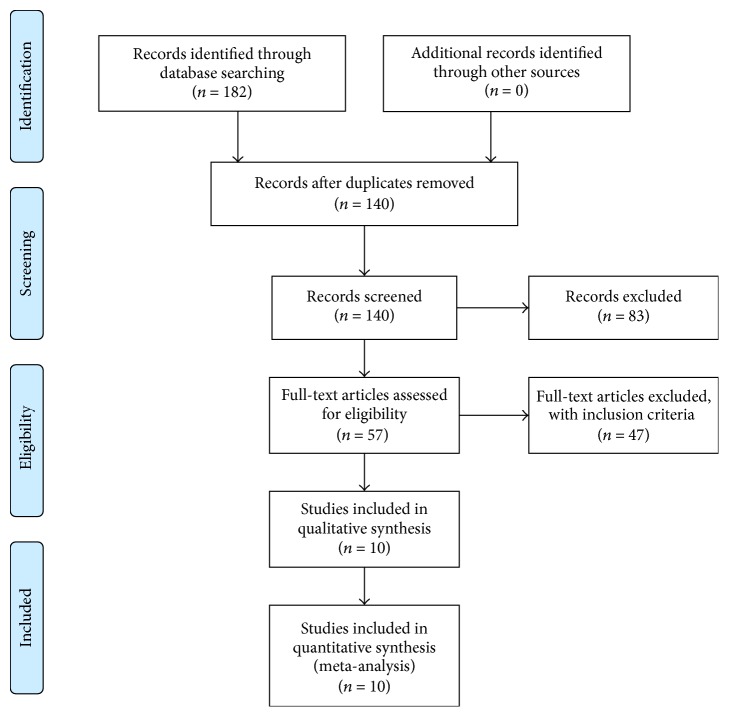
Chart of the article search process.

The studies were included in the meta-analysis if the following criteria were met:

1. the study evaluated the association between glaucoma and ET-1;
2. the report was a case-control study of the disease;
3. healthy subjects were collected as the control group;
4. the plasma ET-1 concentrations in the case subjects and control subjects were measured;
5. it was possible to obtain the full article. A chart of the article search process is presented in Figure 1.

### 2.3. Data Extraction

The articles were reviewed independently by two investigators (Shengjie Li and Aiping Zhang), who searched all the items in the articles. The following characteristics were extracted from each study: (1) name of the first author, (2) publication year, (3) country, (4) ages of the cases and controls, (5) number of cases and controls, and (6) the ET-1 concentration data of the cases and controls.

### 2.4. Statistical Analysis

The statistical analyses were performed using Review Manager 5.2 (the Cochrane Collaboration, Oxford, UK). The heterogeneity of the pooled mean differences was estimated using the *I* ^2^ statistic. The significance of the pooled effect size was determined with the* Z* test. The relative risk was observed using inverse-variance weighted random effects models. The pooled standardized mean difference (SMD) and 95% confidence interval (CI) for ET-1 were calculated for each study. A funnel plot analysis was performed to assess the potential publication bias. We performed a sensitivity analysis to evaluate the stability of the results through a leave-one-out strategy. This method uses sequential omission of individual studies in every comparison to determine whether there is a significant alteration of the combined values. A *p* value of <0.05 was considered statistically significant.

## 3. Results

### 3.1. Characteristics of the Studies

From the initial search strategy, 182 articles were identified, and 172 articles were subsequently excluded (Figure 1). Thus, 10 studies [12–21] were included in the meta-analysis. The characteristics of the studies are listed in Table 1.

### 3.2. Meta-Analysis of the Association of Endothelin-1 with NTG

**Figure 2:**
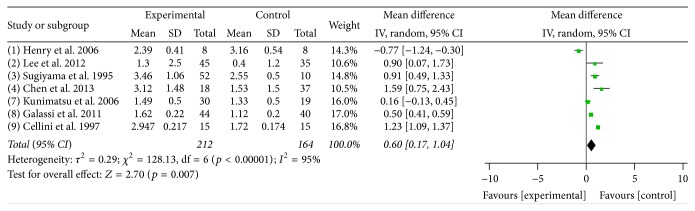
Meta-analysis of the plasma ET-1 levels in the NTG and control groups. A random effects model was used to calculate the mean difference. CI: confidence interval; SD: standard deviation.

Seven studies were included in this meta-analysis, including 212 NTG cases and 164 normal controls. The plasma ET-1 levels were higher in the NTG group than in the control group, with a mean difference of 0.6 pg/mL [*p* = 0.007, 95% CI = 0.17–1.04]; however, there was significant heterogeneity across the 7 studies (*I* ^2^ = 95%, *p* < 0.00001) (Figure 2).

### 3.3. Meta-Analysis of the Association of ET-1 with POAG

**Figure 3:**
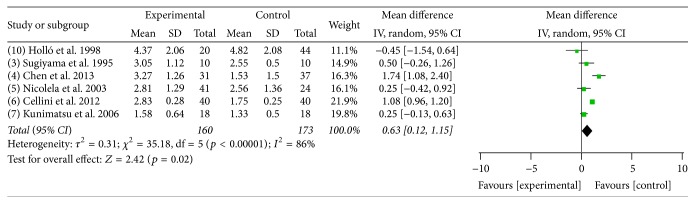
Meta-analysis of the plasma ET-1 levels in the POAG and control groups. A random effects model was used to calculate the mean difference. CI: confidence interval; SD: standard deviation.

Six studies were included in this meta-analysis, including 160 POAG cases and 174 normal controls. The plasma ET-1 levels were higher in the POAG group than in the control group, with a mean difference of 0.63 pg/mL [*p* = 0.02, 95% CI = 0.12–1.15]; however, there was significant heterogeneity across the 6 studies (*I* ^2^ = 86%, *p* < 0.00001) (Figure 3).

### 3.4. Sensitivity Analysis and Publication Bias

**Figure 4:**
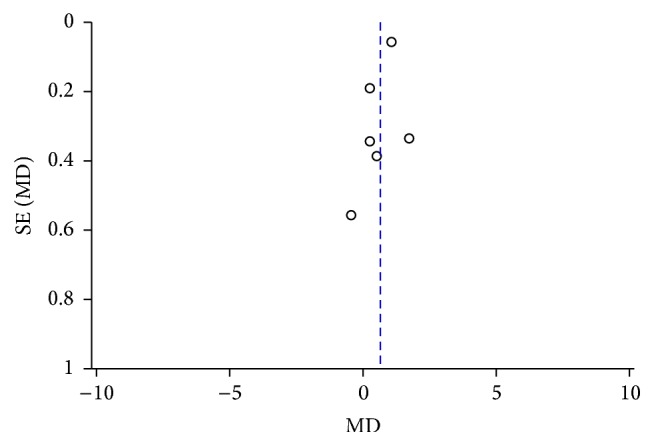
Funnel plot analysis to detect publication bias between the plasma ET-1 level and the risk of POAG.

**Figure 5:**
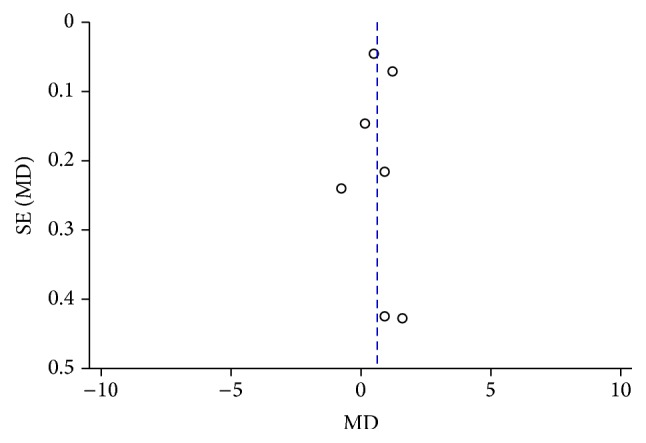
Funnel plot analysis to detect publication bias between the plasma ET-1 level and the risk of NTG.

In the meta-analysis of the association between plasma ET-1 and NTG, the sensitivity analysis revealed that one study had a slight influence on the result. Two studies influenced the meta-analysis results regarding the association between plasma ET-1 and POAG (Table 2). The funnel plot analysis suggested that no publication bias existed (Figures 4 and 5).

## 4. Discussion

Because the relationship of the plasma ET-1 level and the presentation of NTG or POAG remained uncertain, we performed this meta-analysis to clarify the relationship. To the best of our knowledge, this is the first meta-analysis examining the relationship between ET-1 and glaucoma. We found that the ET-1 plasma level was higher in the NTG group than in the control group (*p* = 0.007, 95% CI = 0.17–1.04). Similarly, the plasma ET-1 level was significantly elevated in the POAG group compared with the control group (*p* = 0.02, 95% CI = 0.12–1.15). The results of this meta-analysis indicate that a higher ET-1 plasma level is associated with a significantly increased risk of NTG and POAG.

ET-1 is supposed by some to have association with NTG [12–16]. Lee et al. [14] performed a case-control study and suggested that the systemic levels of ET-1 were significantly higher in the NTG group than in the control group. Similarly, Sugiyama et al. [13] also found that the plasma levels of ET-1 were higher in NTG. In another study conducted in China, Chen et al. [12] also revealed that plasma ET-1 levels were elevated in NTG patients. However, inconsistent results that the ET-1 levels were not significantly different between NTG and control were also reported by Kunimatsu et al. [17] and Henry et al. [18]. Our evaluation in this meta-analysis revealed that ET-1 levels were higher in NTG patients which indicates its positive association with NTG.

Moreover, Chen et al. [12] and Cellini et al. [15] suggested that POAG patients have a higher plasma ET-1 level than normal control also. Nicolela et al. [13, 17, 19] also reported that plasma ET-1 level was relatively higher in POAG than control though the difference was not significant. Only one publication claimed the plasma ET-1 level to be lower in POAG than control [21]. Our study suggested the probability that plasma ET-1 concentrations be elevated in POAG subjects which indicated that it is significantly associated with POAG.

It is of the most interest that in addition to an important role of ET-1 in the pathogenesis of systemic vascular disease, it also may be significant that the plasma level of ET-1 was higher in glaucoma patients. The fact that both NTG and POAG patients have a similarly high ET-1 level might indicate that ET-1 functions in the pathogenesis of both POAG and NTG. Several major population studies and clinical trials have reported the association of cardiovascular disease or hypertension with OAG and have indicated that cardiovascular disease and hypertension increase the risk of OAG [22–24]. The reasons under the relatively surprising observation that glaucoma patients have a higher plasma ET-1 level without presenting with systemic vascular disease remain unclear. However, the following factors might explain this observation: (1) ET-1 might play a role in the pathogenesis of glaucoma and (2) glaucoma patients might have hidden or subclinical vascular disease, which has been suggested in several reports. A recent study revealed that ET-1 dose-dependently enhances cell contraction of the trabecular meshwork (TM) [25]. Another study reported that ET-1 receptor B is expressed in human TM cells [26], and thus an increased ET-1 level might induce ocular hypertension. Karadag et al. showed that the role of ET-1 in glaucoma development involves a mechanism other than increased IOP [27]. Another hypothetical mechanism related to the role of ET-1 in glaucoma is optic nerve ischemia [28]. The eye is frequently involved in vasospastic syndrome in glaucoma patients [29]. The increased level of ET-1 might play an important role in optic nerve ischemia with respect to ET-1 mediated vasoconstriction and the pathogenesis of glaucoma. Consequently, ET-1 might be a good predictive biomarker or a target for pharmacologic intervention.

In this meta-analysis, the increased plasma ET-1 level in POAG and NTG were observed while the heterogeneity should also be considered. A sensitivity analysis was performed by a leave-one-out strategy to further evaluate the stability of the data in the individual studies. A total of three studies [12, 16, 20] had a slight influence on the results, and we considered that the following factors might be responsible. First, the subjects in these studies were of different races and heterogeneity might be introduced as a main source. Treiber et al. [30] and Hartley et al. [31] reported that the African Americans had significantly higher plasma ET-1 levels than European Americans. Moreover it was also reported that the black race exhibited higher plasma levels of ET-1 than the white race [32]. Second, the subjects in these studies were of different socioeconomic status, which might be another reason for heterogeneity. Cooper et al. [33] found that ET-1 levels actually increased in association with different psychosocial burdens in blacks and whites, and plasma ET-1 levels were higher among whites with lower socioeconomic status. Moreover, Hong et al. [34] reported that ET-1 levels were higher in the low and middle social classes as compared with the upper class. Last, the different definitions of NTG and the difference in the ET-1 measurements (in-house RIA versus ELISA, etc.) may also bring some influence on the outcome.

Although a standard search strategy and a thorough computerized search method were applied, certain limitations of our meta-analysis should be considered. First, our meta-analysis included only studies with accessible full-text articles in English. Second, the studies differed widely in the study population characteristics and the measurement techniques. Third, the definitions of NTG across the studies we analyzed were different. Last, only ten eligible studies could be used for the meta-analysis while the number of cases/controls in each study was relatively limited.

In this meta-analysis, we confirmed that NTG and POAG patients have a higher plasma level of ET-1. Consequently, the possible involvement of vascular dysfunction in the pathogenesis of glaucoma and the potential utility of ET-1 as a predictive biomarker might be considered.

## Figures and Tables

**Table 1:** Characteristics of the included studies.

First author	Year	Country	Case			Control		
			*N*	Age (years)	ET-1 (pg/mL)	*N*	Age (years)	ET-1 (pg/mL)
NTG studies								
Henry	2006	England	8	58.12 ± 10.74	2.39 ± 0.41	8	59.37 ± 8.21	3.16 ± 0.54
Lee	2012	Korea	45	53.8 ± 11.8	1.3 ± 2.5	35	52.2 ± 13.9	0.4 ± 1.2
Sugiyama	1995	Japan	52	66.5 ± 11.6	3.46 ± 1.06	10	64.8 ± 19.1	2.55 ± 0.5
Chen	2013	China	18	47.06 ± 14.78	3.12 ± 1.48	37	51.97 ± 17.06	1.53 ± 1.5
Kunimatsu	2006	Japan	30	49.4 ± 8.8	1.49 ± 0.51	19	49.9 ± 5.6	1.33 ± 0.5
Galassi	2011	Italy	44	64.45 ± 6.91	1.62 ± 0.22	40	62.75 ± 7.37	1.12 ± 0.2
Cellini	1997	Italy	15	64.7	2.947 ± 0.217	15	65.8	1.720 ± 0.174

POAG studies								
Sugiyama	1995	Japan	10	64.8 ± 19.1	3.05 ± 1.12	10	64.8 ± 19.1	2.55 ± 0.5
Chen	2013	China	31	48.94 ± 16.77	3.27 ± 1.26	37	51.97 ± 17.06	1.53 ± 1.5
Nicolela	2003	Canada	41	59.5 ± 12.6	2.81 ± 1.29	24	46.9 ± 9.7	2.56 ± 1.36
Cellini	2012	Italy	40	54.5 ± 10.2	2.83 ± 0.28	40	52.9 ± 7.1	1.75 ± 0.25
Kunimatsu	2006	Japan	18	44.7 ± 10.7	1.58 ± 0.64	19	49.9 ± 5.6	1.33 ± 0.5
Holló	1998	Hungary	20	61.5 ± 12.5	4.37 ± 2.06	44	65 ± 12.6	4.82 ± 2.08

*N*: number; data are expressed as mean ± standard deviation (SD).

**Table 2:** Sensitivity analysis by the leave-one-out strategy.

Study omitted	Mean difference	95% CI	*p*
NTG studies			
None	0.60	0.17–1.04	0.007
Henry et al. 2006	0.82	0.40–1.24	0.0001
Lee et al. 2012	0.57	0.10–1.04	0.02
Sugiyama et al. 1995	0.55	0.06–1.04	0.03
Chen et al. 2013	0.49	0.03–0.95	0.04
Kunimatsu et al. 2006	0.69	0.20–1.18	0.006
Galassi et al. 2011	0.64	−0.02–1.31	0.06^Δ^
Cellini et al. 1997	0.47	0.03–0.90	0.03

POAG studies			
None	0.63	0.12–1.15	0.02
Sugiyama et al. 1995	0.65	0.07–1.23	0.03
Chen et al. 2013	0.42	−0.15–0.98	0.15^*∗*^
Nicolela et al. 2003	0.71	0.14–1.27	0.01
Cellini et al. 2012	0.50	−0.14–1.14	0.12^*∗*^
Kunimatsu et al. 2006	0.73	0.17–1.29	0.01
Holló et al. 1998	0.77	0.26–1.228	0.003

CI = confidence interval, ^Δ^ for the influenced meta-analysis results regarding the association between plasma ET-1 and NTG, ^*∗*^ for the influenced meta-analysis results regarding the association between plasma ET-1 and POAG.
